# Caerulomycin A Suppresses Immunity by Inhibiting T Cell Activity

**DOI:** 10.1371/journal.pone.0107051

**Published:** 2014-10-06

**Authors:** Arvind K. Singla, Rama Krishna Gurram, Arun Chauhan, Neeraj Khatri, Rakesh M. Vohra, Ravinder S. Jolly, Javed N. Agrewala

**Affiliations:** 1 Immunology Laboratory, CSIR-Institute of Microbial Technology, Chandigarh, India; 2 Biochemical Engineering Research and Process Development Centre, CSIR-Institute of Microbial Technology, Chandigarh, India; 3 Experimental Animal Facility, CSIR-Institute of Microbial Technology, Chandigarh, India; 4 Department of Chemistry, CSIR-Institute of Microbial Technology, Chandigarh, India; Columbia University, United States of America

## Abstract

**Background:**

Caerulomycin A (CaeA) is a known antifungal and antibiotic agent. Further, CaeA is reported to induce the expansion of regulatory T cell and prolongs the survival of skin allografts in mouse model of transplantation. In the current study, CaeA was purified and characterized from a novel species of actinomycetes, *Actinoalloteichus spitiensis*. The CaeA was identified for its novel immunosuppressive property by inhibiting *in vitro* and *in vivo* function of T cells.

**Methods:**

Isolation, purification and characterization of CaeA were performed using High Performance Flash Chromatography (HPFC), NMR and mass spectrometry techniques. *In vitro* and *in vivo* T cell studies were conducted in mice using flowcytometry, ELISA and thymidine-[methyl-^3^H] incorporation.

**Results:**

CaeA significantly suppressed T cell activation and IFN-γ secretion. Further, it inhibited the T cells function at G1 phase of cell cycle. No apoptosis was noticed by CaeA at a concentration responsible for inducing T cell retardation. Furthermore, the change in the function of B cells but not macrophages was observed. The CaeA as well exhibited substantial inhibitory activity *in vivo*.

**Conclusion:**

This study describes for the first time novel *in vitro* and *in vivo* immunosuppressive function of CaeA on T cells and B cells. CaeA has enough potential to act as a future immunosuppressive drug.

## Introduction

Immunosuppression is the only available therapy for patients undergoing allogeneic organ transplantation. Thus, immunosuppressive drugs play a crucial role in the survival of allogeneic tissue grafts. In the 20^th^ century, many new molecules have been discovered to be used as immunosuppressive agents. Cyclosporine A (CsA), tacrolimus, rapamycin and mycophenolate mofetil (MMF) are the drugs that have proven immunosuppressive activity in patients [1].

Tacrolimus and CsA are the calcineurin inhibitors (CNI) [2]. The introduction of calcineurin inhibitors was a revolutionary event in the history of transplantation [3]. It not only dramatically improves the outcome of graft acceptances, but also transplantation of heart, liver and pancreas became possible. Besides these advantages, CNI are also associated with adverse side effects. Those include nephrotoxicity, malignancy, and hypertension [4], [5], [6]. This compromises the overall benefits of the drug during long-term application. The immunosuppressive agents MMF and sirolimus have been shown to be well tolerated and effective alternative for CNI [7], [8]. Recent studies revealed that these drugs have severe side-effects during long-term clinical applications [9], [10]. Consequently, invention of novel immunosuppressive molecules with better mode of action and minimum side-effects are urgently desired for patients undergoing long-term treatment.

Most of the immunosuppressive drugs are discovered from secondary metabolites of microorganisms [11]–[13]. In search of a novel immunosuppressive drug, we screened various extracts secreted by microorganisms collected from different niches of India. Interestingly, we discovered bioactive compound produced by a new strain of actinomycetes. This was named as *Actinoalloteichus spitiensis (A. spitiensis)* [14]. The compound exhibited potent immunosuppressive property on T cells. The NMR data revealed that the active compound was Caerulomycin A (CaeA).

Recently, we have described a novel role of CaeA in inducing the generation of regulatory T cells (Tregs) [15]. Further, it suppressed the mixed lymphocyte reaction and prolonged the survival of skin allografts in experimental model of transplantation [16]. In the current study, we report that CaeA effectively inhibited *in vitro* and *in vivo* activity of T cells and B cells. Both these cells play an imperative role in the graft rejection. Therefore, CaeA may have an important application as an immunosuppressive drug in the future.

## Materials and Methods

### Mice

Inbred female BALB/c, C57BL/6J and C3He mice, 6–8 weeks old were obtained from the institute's animal facility. The animals were housed under normal conditions and food and water were available *ad libitum*.

### Ethics statement

The animal study was approved by Institutional Animal Ethical Committee (IAEC) of Institute of Microbial Technology, Chandigarh. These experiments were performed according to National Regulatory Guidelines issued by Committee for the Purpose of Experiments on Animals (CPCSEA), Ministry of Environment and forest, Govt. of India.

### Chemicals and reagents

All cytokines and Abs used for ELISA and flowcytometry were procured from Becton Dickinson (Franklin Lakes, NJ), or otherwise mentioned. Growth media components were purchased from Hi-media (Mumbai, India) and Difco (Detroit, MI). Sodium acetate, lysozyme, proteinase K, RNase A, saponin, paraformaldehyde, CsA and other fine chemicals were purchased from SIGMA-ALDRICH (St. Louis, MO). CaeA was either procured from LKT Laboratories (St. Paul, MN) or purified from actinomycetes *A. spitiensis*. Aluminium sheets pre-coated with silica gel 60 F_254_ were from Merck (Darmstadt, Germany). Fetal calf serum (FCS) was purchased from Harlan Sera Lab (Crawley Down, Great Britain). RPMI 1640, DMEM, HBSS and APO-BrdU TUNEL assay kit were bought from Invitrogen (Carlsbad, CA). L-glutamine, L-pyruvate, penicillin, concanavalin A and streptomycin were procured from Serva (Heidelberg, Germany). Thymidine-[methyl-^3^H] was the product of Amersham Pharmacia Biotech AB (Uppsala, Sweden).

### Medium

Cells were cultured in RPMI 1640 or DMEM medium supplemented with 10% FCS, L-glutamine (2 mM), penicillin (50 µg/ml), streptomycin (50 µg/ml), HEPES (100 mM) and 2-mercaptoethanol (0.05 mM).

### Fermentation of CaeA

CaeA was isolated from *Actinoalloteichus spitiensis* sp. nov. [14]. The stock culture of the organism was inoculated into a 500 ml Erlenmeyer flask containing 100 ml of the seed medium composed of (per litre): glucose: 5.4 g, yeast extract: 4.8 g, malt extract: 8.5 g and CaCO_3_: 3 g (pH- 8.0). After incubation at 28°C for 48 h on a rotary shaker at 220 rpm, vegetative culture was transferred at a rate of 5% v/v into five 1L flasks containing 200 ml of medium. The seed culture thus obtained was transferred into a 20L fermenter containing 14L of seed medium. Fermentation was carried out at 28°C, 300–350 rpm agitation and 1 vvm of aeration. The growth is represented in terms of packed mycelial volume (PMV) measurement. CaeA production was monitored by colorimetric quantification method. In 2 ml of culture broth, 2 ml of 10 mM ferrous ammonium sulphate solution was added followed by 1 ml of 1% Na_2_CO_3_. Extraction of this solution was done with 5 ml of n-butanol. The absorbance of clear solution was read at 532 nm and finally compared with the standard CaeA. Residual sugar was determined by the 3, 5-dinitrosalicylic acid (DNS) method using glucose as standard [17].

### Purification and structure elucidation of CaeA

After fermentation, ethyl acetate extraction of the culture broth, and concentration *in vacuo* was done to obtain semisolid crude residue. The compound was purified by using HPFC (Horizon HPFC system, Biotage, San Francisco, CA) on silica gel (32–63 µM, 60 Å). Column was eluted with benzene∶acetone (3∶1). The purity of the compound was checked by TLC and HPLC. For structure elucidation, standard techniques like Proton Magnetic Resonance Spectrometry (^1^HNMR), ^13^C NMR Spectrometry (^13^C NMR), Mass Spectrometry (MS) and Infrared Spectrometry (FT-IR) were used.

### Estimation of cytokines

The cytokines IL-2, IL-5, IL-10, TGF-β and IFN-γ in the culture SNs and serum were estimated by sandwich ELISA, according to the manufacturer's instruction. Briefly, 96w ELISA plates were coated (50 µl/well) with anti-IL-2 (4 µg/ml), anti-IL-5 (6 µg/ml), anti-IL-10 (6 µg/ml), anti-TGF-β (4 µg/ml) and anti-IFN-γ (4 µg/ml) Abs, in phosphate buffer (pH 9.2, pH 6 for IL-10) and incubated for 12 h at 4°C. Later, the plates were blocked with BSA (1%, 100 µl/well). ELISA plates were incubated with culture SNs (50 µl/well) and their respective recombinant cytokine standards (40–2000 pg/ml) for 12 h at 4°C. These plates were treated with their corresponding biotin conjugated secondary Abs, followed by streptavidin-HRP. Plates were developed using substrate H_2_O_2_ and chromogenic agent OPD (o-phenylenediamine). The reaction was stopped by mixing equal volume of 7% H_2_SO_4_. Optical density (OD) of the color developed was measured at 495 nm. Usual steps of incubations and washings using PBS/Tween-20 (0.05%) were followed at each step. The level of cytokines was estimated by plotting standard curve using recombinant cytokines. Values are expressed as pg/ml. The same procedure was followed for measuring isotypes IgG1 and IgG2a in the serum using appropriate reagents.

### Expression of CD69 on the surface of CD4 T cells by flowcytometry

The cells were harvested from the cultures and Fc receptor was blocked with anti-CD16 Ab, followed by staining with fluorochrome conjugated anti-CD4 and CD69 Abs and their isotype-matched controls for 30 min at 4°C. Usual steps of washings were performed at each stage using flowcytometry buffer (PBS 1X+2% FCS). Finally, cells were fixed in PBS containing paraformaldehyde (1%). The cells were acquired utilizing BD FACSCalibur, and analysis was performed with the help of BD FACSDiva software.

### Cell cytotoxicity assay

CD4 T cells were stimulated with anti-CD3 and CD28 Abs. The cultures were treated with CaeA (0.15–0.62 µM) for 48 h. Later, cytotoxicity of drug treatment was measured by TUNEL assay (APO-BrdU TUNEL assay kit), according to the manufacturer's instruction. Briefly, cells were harvested and media components were removed by washing with excess quantity of PBS, followed by fixing them in paraformaldehyde (1%). Later, the cells were incubated in ethanol (70%) at −20°C for 18 h. The nicked ends of DNA in apoptotic cells were labelled with BrdU for 60 min at 37°C followed by detection with Alexa fluor 488 labelled anti-BrdU Abs. These cells were counter stained with PI/RNaseA staining buffer. The apoptotic human lymphoma cell line recommended by assay kit was used as positive control. Cells were acquired in BD FACSCalibur and analysis was performed by BD FACSDiva software.

### Proliferation and reversibility assay of mitogen stimulated T cells cultured with CaeA

ConA (2 µg/ml) stimulated splenocytes (SPC) (2×10^5^/well) from BALB/c mice were cultured with increasing concentrations of CaeA (0.04–0.31 µM) in triplicate in ‘U’ bottomed 96w microtitre tissue culture plate in RPMI/FCS-10% (200 µl). In control cultures, cells were incubated with only ConA. After 48 h, the cultures were pulsed with thymidine-[methyl-^3^H] (0.5 µCi) and harvested 16 h later by automatic 96 well plate cell harvester (Tomtech, Hamden, CT). Radioactivity incorporated was measured by liquid scintillation counting (1450 Micro Beta TriLux Microplate counter, PerkinElmer, Waltham, MA).

In parallel, the similar cultures were set as indicated in the case of T cell proliferation. After 48 h, cells (2×10^5^/well) were washed 3× with 1× PBS and restimulated with ConA (2 µg/ml) for 48 h. The proliferation was measured as mentioned above. The proliferation was directly proportional to the radioactivity incorporated and data were expressed as counts per minute (cpm).

### *In vivo* toxicity assay

The *in vivo* toxicity was performed by feeding the mice with different doses (0.25–25 mg/kg body weight) of CaeA for 14 days. Throughout the study various parameters like body weight, food and water intake was monitored. At the end of the study, mice were sacrificed and weight of various organs (liver, heart, kidney and spleen) was measured. Organs of animals were also examined for abnormalities by gross pathology. The *in vivo* toxicity experiments were performed at the National Institute of Pharmaceutical Education and Research, Mohali, India.

### Cell cycle analysis

Jurkat cells were synchronized by serum starvation overnight and then exposed to CaeA and CsA (0.15 µM) for 24 h and 48 h. Thereafter, cells were washed, and fixed with ethanol (70%). Later, ethanol was removed and cells were suspended directly in staining solution (propidium iodide: 25 µg/ml, RNase A: 0.1 mg/ml, TritonX-100: 0.05% in PBS), and incubated at RT for 20 min in darkness. Immediately, cells were acquired using BD FACSCalibur and data were analyzed with the help of ModFit LT software.

### *In vivo* T cell response

The emulsion of antigen ovalbumin (OVA) (2 mg/ml in PBS) was prepared in equal volume of complete Freund's adjuvant. Emulsion (100 µl/mouse) was injected intraperitoneally (i.p.) in BALB/c mice. After 7 days, the animals were given a booster dose of antigen (50 µl/mouse) emulsified in incomplete Freund's adjuvant. Different sets of experimental and control groups (5 mice/group) of animals were orally fed CaeA (2.5 mg/kg body weight) daily for 7 days. The control groups were administered CMC and PBS. Later, mice were sacrificed and SPC (2×10^6^/ml) were pooled and *in vitro* cultured with increasing concentrations of OVA. After 48 h, these cultures were pulsed with thymidine-[methyl-^3^H] (0.5 µCi/ml) for 16 h. Later, cells were harvested and radioactivity incorporated was measured by liquid scintillation counting and expressed as cpm.

### Statistical analysis

Statistical analysis was performed using GraphPad Prism software. The differences between two values were compared by Unpaired Student's ‘t’ test two tailed. In case of comparison between two groups, two-way ANOVA was used for measuring significant difference.

## Results

### Fermentation, purification and structural elucidation of CaeA

A time course fermentation analysis plot for organism *A. spitiensis* (Figure S1 A, B); showing increase in the growth and CaeA production and decrease in residual sugar over time. Maximum growth of the strain was observed after 24 h and CaeA yield peaked near 72 h. Broth obtained after 72 h was extracted with ethyl acetate and concentrated *in vacuo*. HPFC was performed to purify CaeA using benzene∶acetone (3∶1). TLC showing the purification profile of CaeA (Figure S1 C). Further, based on NMR, IR and mass spectral data, the identified compound was CaeA (Figure S2). The result corresponds well with the already reported data on CaeA [18]–[20]. Structure was further confirmed (^1^HNMR) by converting CaeA into methyl ether by its reaction with methyl iodide in the presence of anhydrous potassium carbonate in anhydrous acetone (Table S1).

### Induction of the suppression of T cell response by CaeA

**Figure 1:**
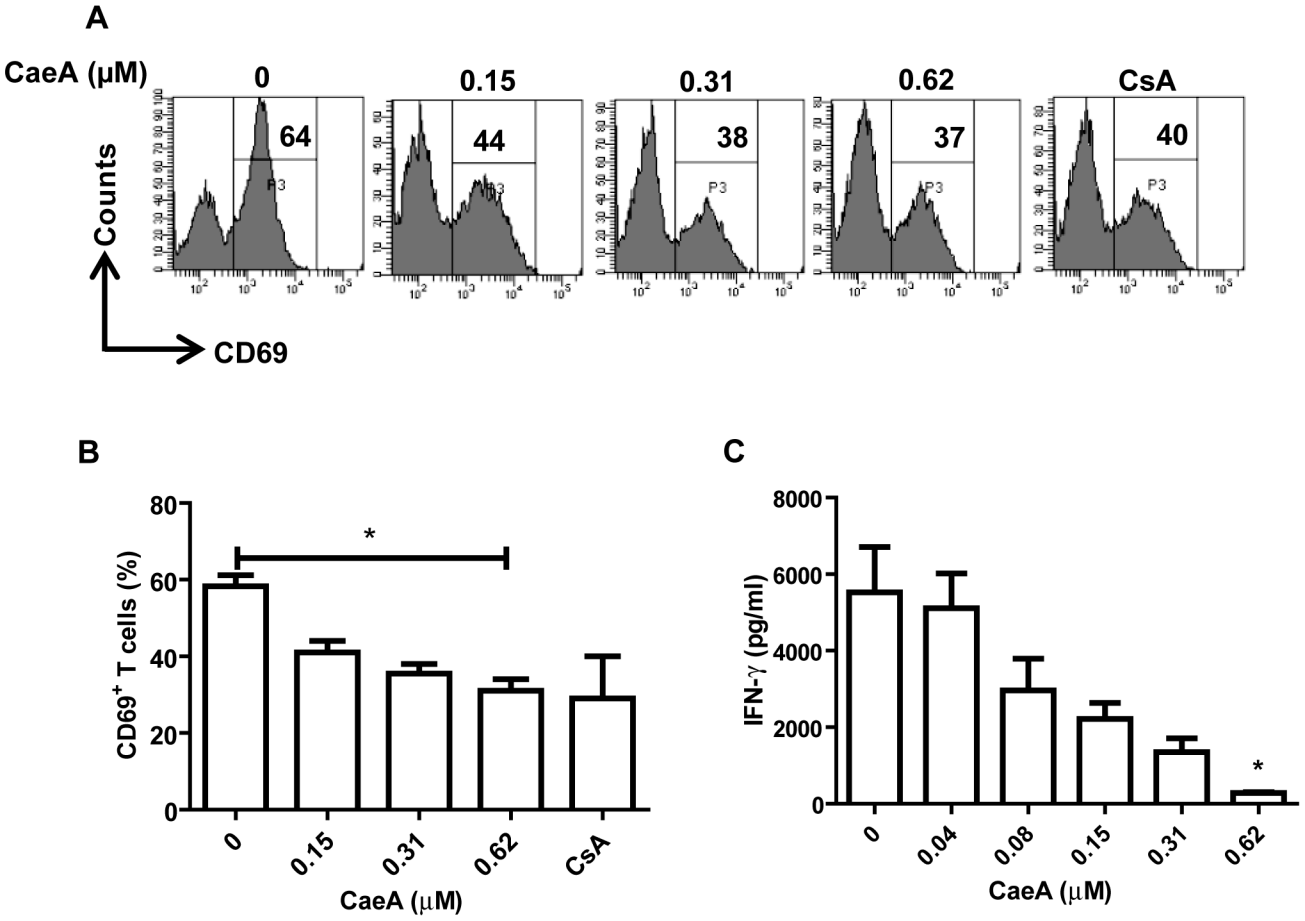
CaeA suppresses T cell activation and IFN-γ secretion. CD4 T cells stimulated with anti-CD3 and CD28 Abs were incubated with indicated concentrations of CaeA for 48 h. (A) Flowcytometric histogram represents CD69 expression. Value in the inset indicates percentage; (B) bar diagram depicts decreased percentage of CD4 T cells expressing CD69; (C) ELISA data depicted as pg/ml shows decrease in IFN-γ secretion by CaeA. ‘*’ represents p<0.05. Results are representative of three independent experiments.

In the initial phase of the study, we checked the immunosuppressive activity of CaeA on T cells on the expression of the activation marker CD69, which is upregulated during T cell activation [21]. Interestingly, we observed significant (p<0.05) decline in the display of CD69 by CaeA (Figure 1 A, B). The change was seen in a dose dependent manner. CsA, an immunosuppressive drug, known to inhibit CD69 expression on the activated T cells was used as a positive control [22]. Since CaeA considerably retarded CD69 expression we next evaluated its influence on the IFN-γ secretion. We found that CaeA markedly (p<0.05) decreased the secretion of IFN-γ (Figure 1 C). The inhibition in the IFN-γ secretion by CaeA was noticed in a dose dependent fashion. Whereas, no change in the IFN-γ level was noted in the control cultures incubated with DMSO alone. These results may provide some insight into the mode of action of immunosuppression by CaeA by downregulating the expression of CD69. The specificity of CaeA on T cell activity was established by analyzing LPS induced macrophage activation. CaeA treatment does not affect the expression of CD86 and nitric oxide (NO) and proinflammatory cytokines IL-6 and TNF-α release from macrophage (Figure S3).

### CaeA inhibits IL-10 but not TGF-β secretion

**Figure 2:**
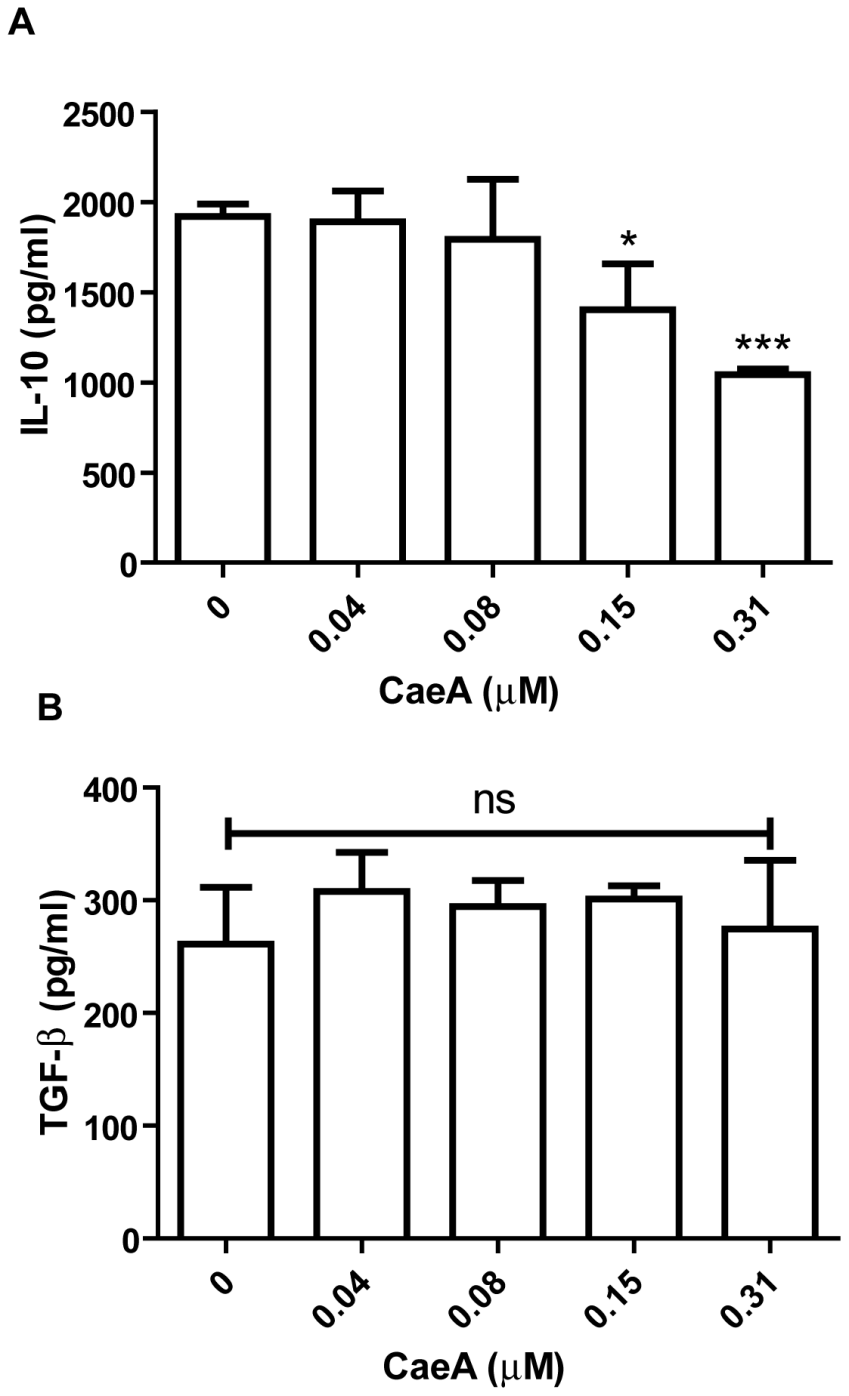
CaeA inhibits the production of IL-10. CD4 T cells stimulated with anti-CD3 and CD28 Abs were cultured with different concentrations of CaeA for 48 h. Later, the cytokines level was estimated by ELISA. Bar diagrams represent mean+SD of (A) IL-10; (B) TGF-β estimated in the culture supernatants. ‘*’, ‘***’ denote p<0.05, p<0.0001, respectively. Results shown are of two independent experiments.

CaeA significantly (p<0.0001) retarded the yield of IL-10 by CD4 T cells activated by anti-CD3 and CD28 Abs (Figure 2A). No change was noted in the case of TGF-β release (Figure 2B). These results indicate that CaeA mediated inhibition of the T cell activation and IFN-γ secretion is not due to the involvement of either IL-10 or TGF-β.

### Inhibition of T cell response by CaeA is not due to cytotoxicity

**Figure 3:**
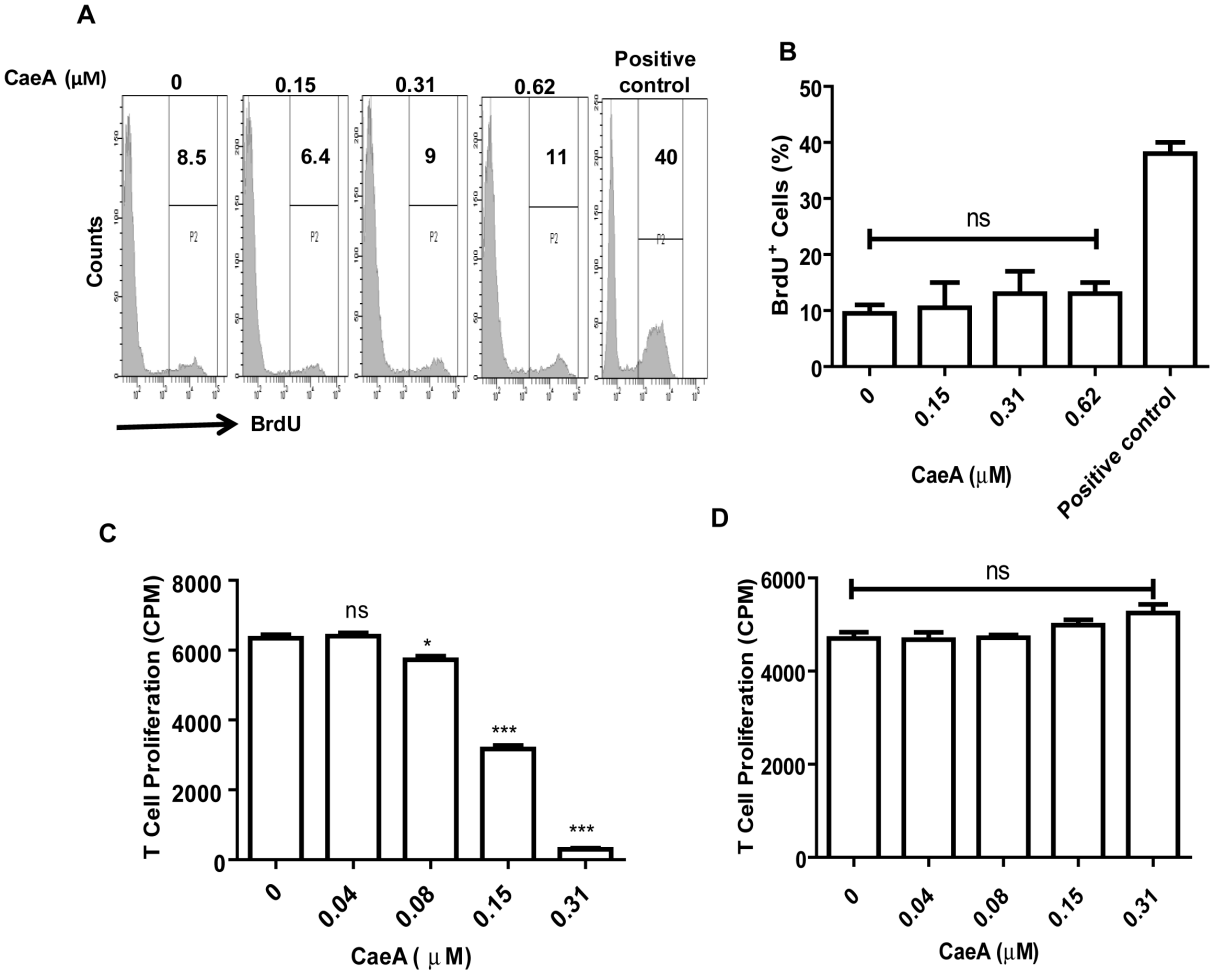
CaeA is not toxic to CD4 T cells. Purified CD4 T cells stimulated with anti-CD3 and CD28 Abs were cultured with different concentrations of CaeA for 48 h. Later, frequency of CD4 T cells undergoing apoptosis was analyzed. (A) Flowcytometric histograms represent BrdU incorporation (APO-BrdU TUNEL assay) of cells undergoing apoptosis. Diagram also shows positive control; (B) bar diagram denotes flowcytometry data of the percentage of cells undergoing apoptosis. Value in the inset of histogram depicts the percentage of cells; (C) inhibition in the proliferation of T cells by different doses of CaeA, monitored by thymidine-[methyl-^3^H] incorporation and expressed as cpm; (D) bar diagram signifies the reversibility in T cell proliferation after the removal of CaeA. Results expressed as percentage (A); mean±SE (B-D) are from three independent experiments. ‘*’, ‘***’ specify p<0.05, p<0.0001, respectively.

We were concerned that CaeA mediated inhibition of IFN-γ secretion and CD69 expression was due to immunosuppression and not cytotoxicity. Hence, we did TUNEL assay. The results confirmed that CaeA is not toxic to T cells, as evidenced by lesser percentage of BrdU^+^ cells by flowcytometry data (Figure 3A, B). The studies on T cells reversibility after removal of CaeA also supported that CaeA is not toxic at the concentration, which is inducing immunosuppression. The ConA stimulated T cell proliferation was significantly (p<0.0001) suppressed by CaeA treatment (Figure 3C). However, removal of CaeA after 48 h of treatment from the cultures could still revert back T cells to normal state, as evidenced by proliferation upon activation with ConA (Figure 3D).

### CaeA induced the suppression of T cells by arresting the cell cycle at G_1_ phase

**Figure 4:**
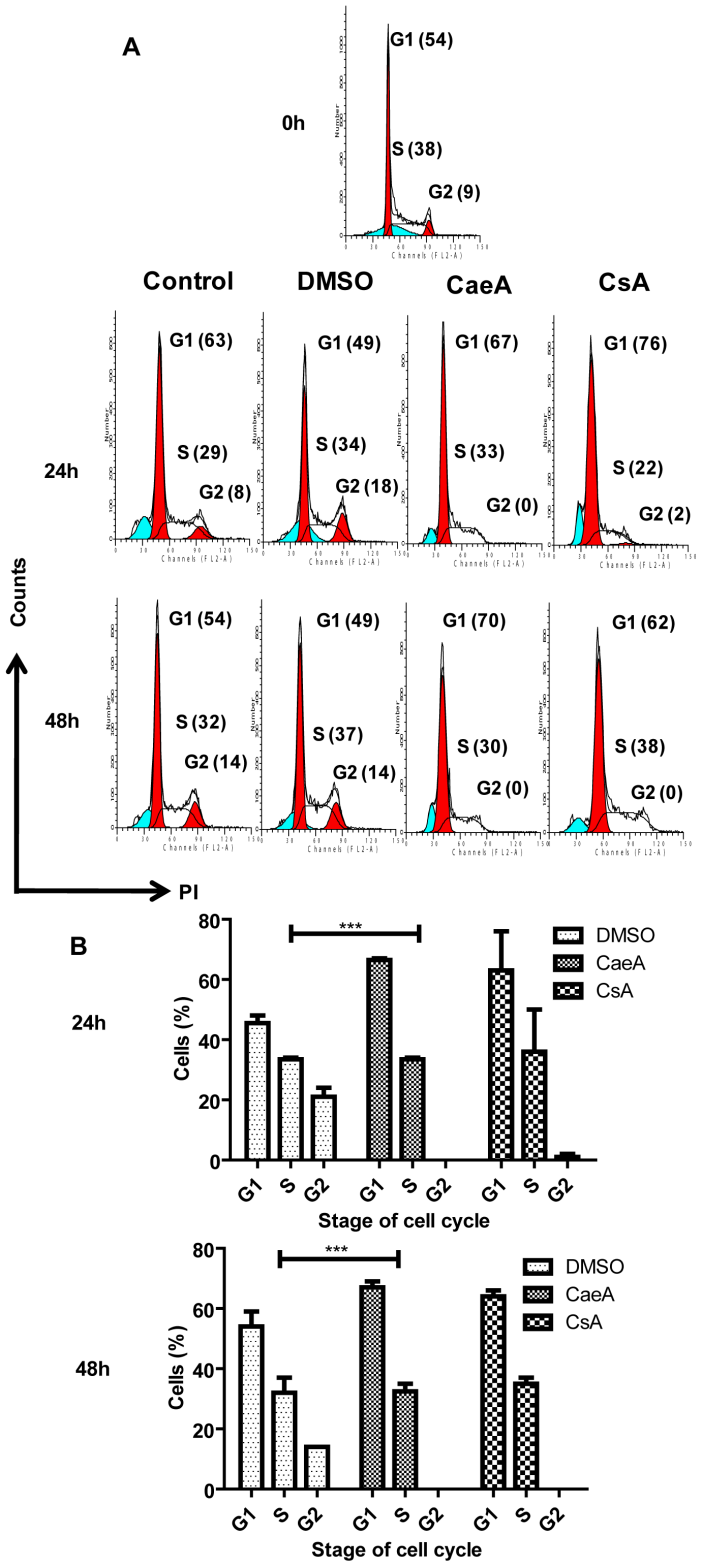
CaeA induces cell cycle arrest at G1 phase. Synchronized T cells (Jurkat) were incubated with CaeA for 24 h and 48 h and cell cycle analysis was performed. (A, B) Flowcytometry and histograms depict the percentage of cells at different stages (G1, G2 and S) of cells cycle. Values in the (A) inset of the histograms represent percentage of cells; (B) bar diagrams as mean±SE of the data obtained from flowcytometric analysis for cell cycle arrest at 24 h and 48 h. Results are representative of two independent experiments. ‘***’ indicates p<0.0001.

We observed that CaeA inhibited T cell proliferation by arresting the cell cycle at G1 phase (Figure 4A, B), as documented by the increase in PI incorporation (70%), compared to vehicle (DMSO) control (49%) at 48 h. The concentration 0.15 µM of CaeA was chosen in the experiment, since it induced significant decline in the proliferation of T cells (Figure 3C). An equimolar concentration of CsA was used as a positive control.

### CaeA induced the *in vivo* suppression of T cell proliferation

**Figure 5:**
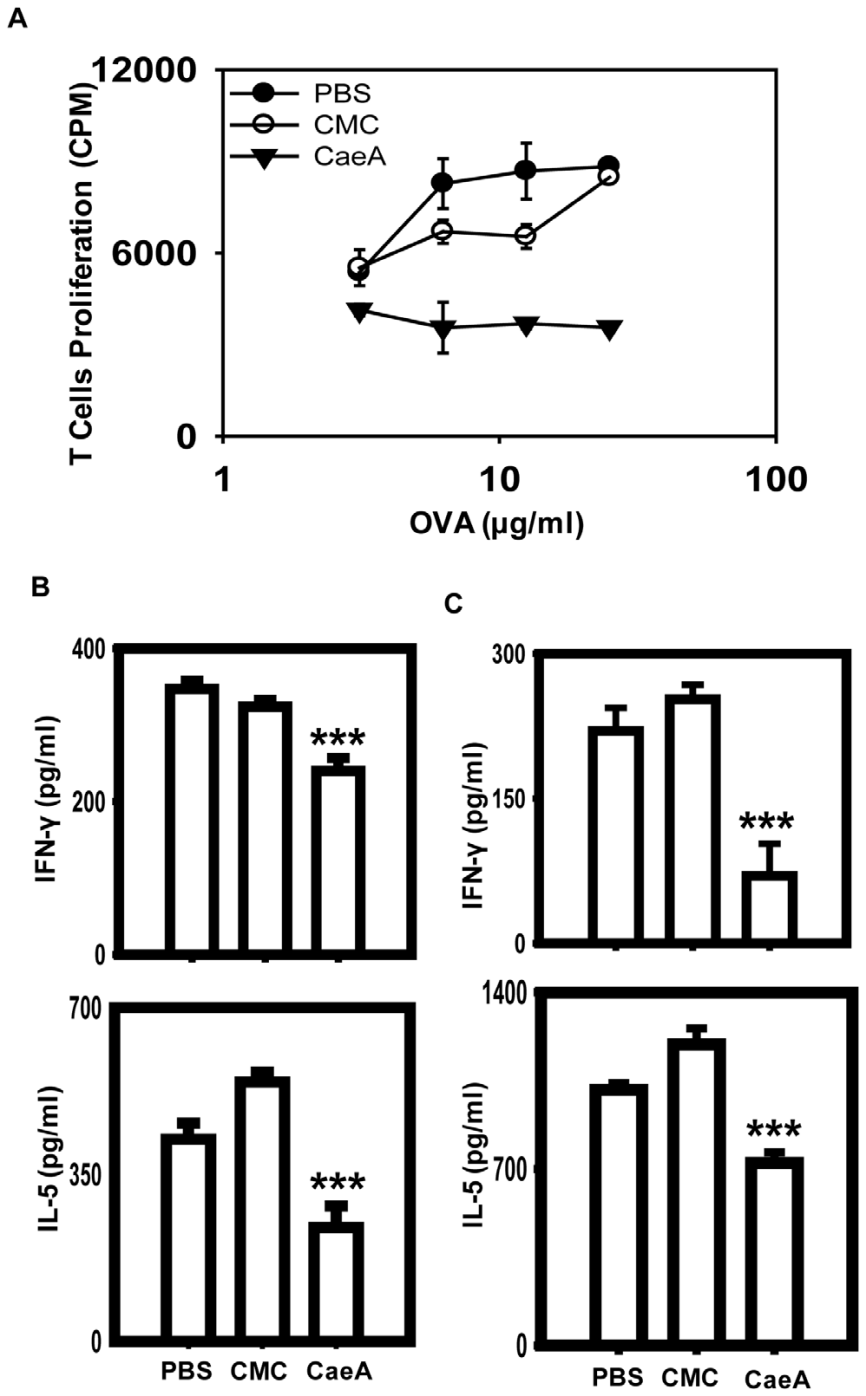
CaeA treatment inhibits *in vivo* T cell activation. Animals immunized with antigen were treated with CaeA and suppression in the activation of T cells was examined. (A) Line diagrams designate the suppression in antigen specific T cell proliferation, measured by thymidine-[methyl-^3^H] incorporation; (B, C) bar diagram refers to the estimation of IFN-γ and IL-5 (pg/ml) by ELISA in the (B) serum and (C) culture SNs. Results are representative of three independent experiments. Error bars indicate mean±SD. ‘***’ specifies p<0.0001.

**Figure 6:**
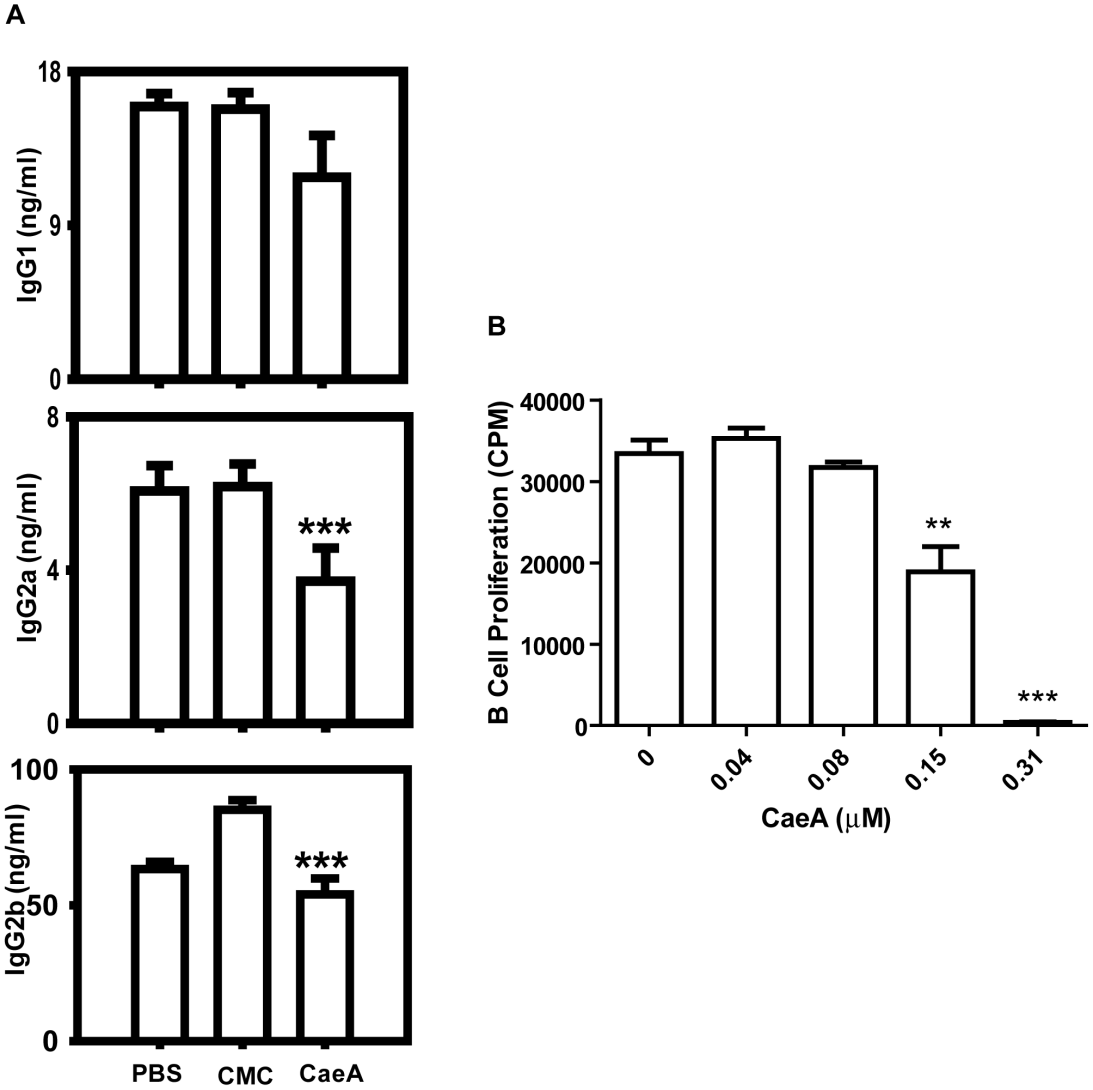
CaeA retards the *in vivo* function of B cells. Antigen primed mice were treated with CaeA and the inhibition in the function of B cells was monitored. (A) Bar diagram denotes yield of IgG1, IgG2a and IgG2b isotypes, estimated by ELISA in the serum; (B) The proliferation B cells treated *in vitro* with doses of CaeA was examined by radioactivity incorporation and expressed as cpm. Results are representative of three independent experiments. Error bars indicate mean±SD. ‘**, ‘***’ specify p<0.005, p<0.0001, respectively.

Suppression of the activation of antigen reactive T cells and B cells has therapeutic implication in inhibiting the undesired immune response [23], [24]. Hence, we next wanted to check whether CaeA can suppress the activation of antigen reactive T cells and B cells *in vivo*. Our experimental results showed that T cells obtained from antigen-primed mice, which were orally fed CaeA for 7 d, exhibited substantial inhibition (p<0.0001) in the proliferation (Figure 5A). Further, significant decrease (p<0.0001) was seen in the serum levels of IFN-γ and IL-5 (Figure 5B), as well as in the SNs collected from the cells cultured *in vitro* with antigen and CaeA (Figure 5C). Furthermore, noticeable retardation (p<0.0001) in the level of serum IgG1, IgG2a and IgG2b was measured in the CaeA treated mice (Figure 6A). We also observed substantial (p<0.0001) decline in the proliferation of LPS stimulated B cells *in vitro* and treated with CaeA (Figure 6B). No change was observed in the control groups administered with either PBS or CMC (Figure 5, 6). Since proliferation of both T cells and B cells is inhibited by the CaeA, we conjecture that the reduced Ab titer was because of the inhibitory effect on both the cells.

We also monitored the *in vivo* toxicity of CaeA on mice by recording the change in different parameters (mortality, body weight, gross pathology, food and water intake and weight of organs) for 14 days. Except minor change in liver weight, CaeA treatment does not induce any significant variation in any of the parameters studied (Figure S4).

### CaeA mode of action is different from calcineurin inhibitors

**Figure 7:**
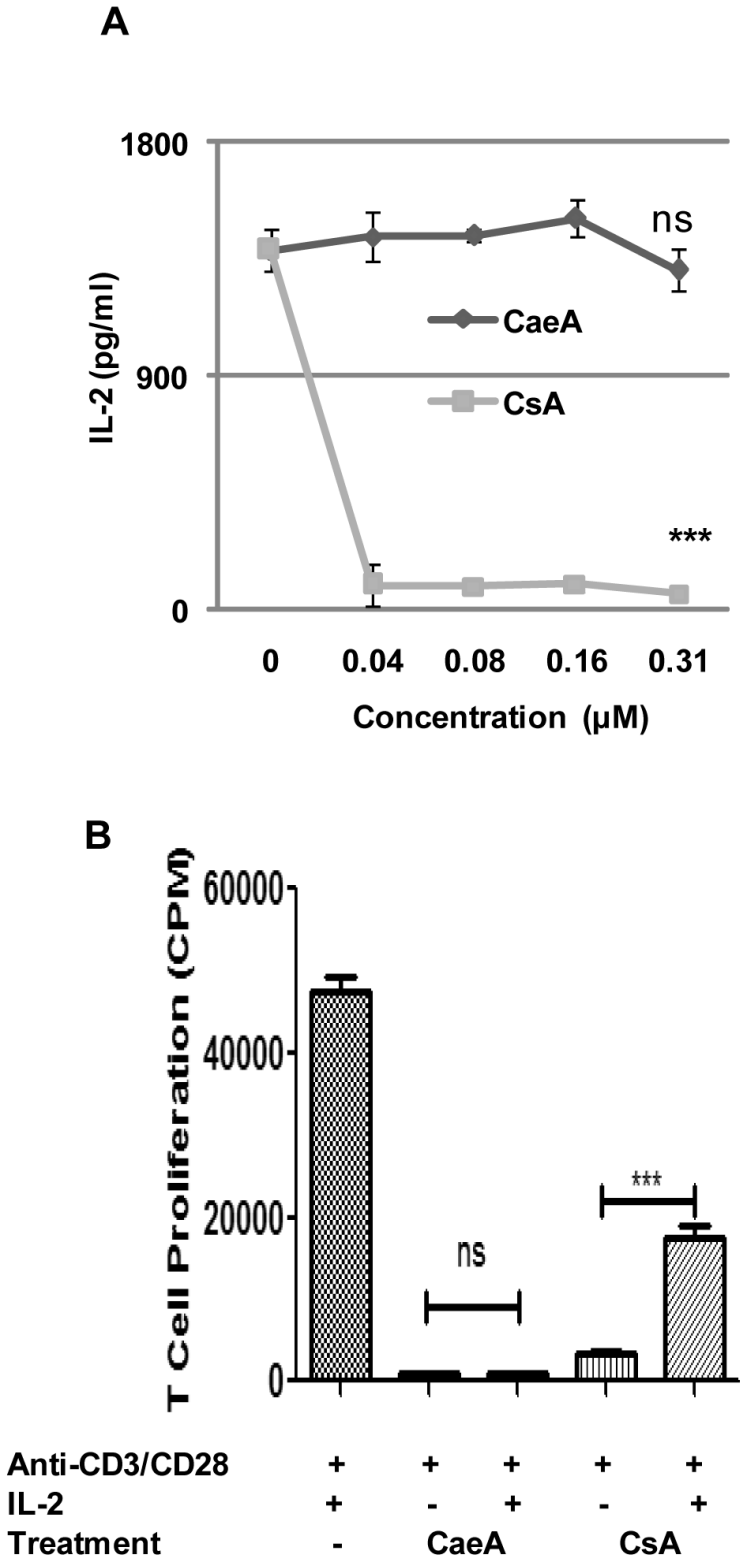
CaeA mediated immunosuppression is different from calcineurin inhibitors. CD4 T cells stimulated with anti-CD3 and CD28 Abs were incubated with CaeA and CsA and monitored for (A) IL-2 secretion; (B) modulation of the proliferation by CaeA and CsA in the presence or absence of IL-2. Data shown as mean±SE are representative of 3 independent experiments. ‘***’ signify p<0.0001 and ns as non-significant.

It was important for us to monitor whether the mode of immunosuppressive activity of CaeA was same as observed in the case of CsA. CsA exerts its inhibitory action through calcineurin pathway [25]. Interestingly, inhibition in the secretion of IL-2 was seen with CsA but not CaeA (Figure 7A). IL-2 secretion is an indicator of the activation of calcineurin pathway (25). Further, we could rescue the inhibition in the proliferation induced by CsA by addition of exogenous IL-2 (Figure 7B). In contrast, no change was measured in the case of CaeA (0.15 µM). Thus, the results depicted that the mechanism of action of CaeA is distinct from CsA.

## Discussion

Actinomycetes are one of the most competent microorganisms capable of producing secondary metabolites with multiple biological activities like antifungal, immunosuppressive, antibacterial, antiviral, anticancer and anti-inflammatory, etc. [26]. CaeA used in this study was identified, isolated and purified from a novel species *A. spitiensis* during screening of bioactive molecules.

In allogeneic organ transplantation, the immune system of recipient will recognize the transplanted organ as foreign antigen and elicit immune response. The professional antigen presenting cells (DCs) from donor graft will migrate to the secondary lymphoid organs. In the secondary lymphoid organs these DCs will activate the naïve T cells to become effector T cells [27]. T cells play a fundamental role in the initiation and regulation of immune responses [28]. The role of T cells is well established in autoimmune diseases and graft rejection [29], [30]. T cells undergone activation will emerge from lymph nodes and infiltrate the implanted organ and orchestrate the process of rejection. The effector T cells generated against the alloantigen will undergo activation and produce IFN-γ. Immune cells like macrophages, neutrophils and B cells will respond to the IFN-γ, leading to the tissue destruction. B cells after interacting with T cells will secrete the antibody against allograft and are also involved in the graft rejection. Therefore, it is crucial to suppress the activation of T cells.

The undesired immune response elicited against the engrafted tissue is suppressed with the help of immunosuppressive drugs. Existing treatment to suppress the alloreactivity largely depends on drugs like azathioprine, mycophenolate mofetil, CsA, tacrolimus, rapamycin, corticosteroids, etc. [1]. These drugs are toxic and associated with severe side-effects. Hence, there exists a necessity to search new drugs, which can efficiently exert immunosuppression without inflicting much harm to the patients. In this connection we have earlier demonstrated the suppressive role CaeA by inducing the generation of Tregs [15]. Further, we have also shown that CaeA suppressed the activation of allogeneic T cells and substantially prolonged the survival of mouse skin allografts [16]. In the present study we attempted to analyse the immunosuppressive activity of CaeA on polyclonal and antigen reactive T cell and B cell populations. Following major results have emerged signifying that CaeA i) downregulates the expression of CD69; ii) suppresses the secretion of IL-5, IL-10, IFN-γ; iii) is not toxic at the optimum dose; iv) arrests the T cell cycle at G1 phase; v) impedes the function of antigen reactive T cells and B cells; vi) mediates immunosuppression through mechanism distinct from calcineurin inhibitors.

Interestingly, CaeA suppresses CD69 expression on activated T cells. The generation of immune response begins with T cell activation [31]. The activated T cells upregulate the expression of CD69 on their surface [21]. Recent studies suggest that CD69 negatively regulate the Tregs by favouring effector T cell differentiation [32]. Therefore, inhibition of T cell activation is a potential approach to treat graft rejection and autoimmune diseases [33]–[35].

On the basis of cytokines profile, CD4 T cells can be subdivided into Th1 and Th2 cells. Th1 cells mainly produce IL-2 and IFN-γ and are responsible for cell-mediated immunity. In contrast, Th2 cells chiefly secrete IL-4 and IL-5 and are responsible for humoral immunity [36]. Further, Th1 dominated immune responses are often associated with inflammation and tissue injury; because IFN-γ recruits and activates macrophages and neutrophils. The typical inflammatory reaction is delayed type hypersensitivity, which is also Th1-mediated and accompanied by host tissue damage [37], [38]. IFN-γ also antagonize the generation of Tregs [39]. We observed that CaeA notably suppressed the IFN-γ secretion from activated T cells.

After receiving antigen stimulation, T cells undergo clonal expansion. Rapamycin is a very well-known immunosuppressive drug, which diminishes the T cell proliferation by preventing the cells to enter from S to G1 phase [40]. CaeA exhibited homology with rapamycin in arresting the T cells at G1 phase of the cell cycle. Likewise, CaeA and rapamycin also induced the generation of Tregs [15], [41]. Recently, we have demonstrated that CaeA considerably up-regulated the expression of Foxp3 on CD4 T cells and decreased the frequency of Th1 and Th17 cells. The possible mechanism identified indicated that CaeA obstructed IFN-γ-induced STAT1 signaling by enhancing the display of SOCS1 and increased TGF-β mediated Smad3 function. Consequently, supported the generation of Tregs [15].

The *in vitro* findings of immunosuppressive influence of CaeA on T cells gave considerable impetus to our confidence when similar results were replicated *in vivo*, as well. The decrease in the proliferation of T cells in the CaeA treated group of animals upon antigen challenge may be due to induction of Tregs by CaeA, as reported in our recent observation [15]. The role of antibodies has also been implicated in the graft rejection and many autoimmune diseases [42], [43]. It is also very well established fact that when Th1 and Th2 cells interact with B cells, they mainly produce IgG2a and IgG1, respectively. Consequently, inhibition in the secretion of IgG1 and IgG2a *in vivo* further authenticates our observation that CaeA suppresses the activation of Th2 and Th1 cells. It is worth to mention here that CaeA suppressed the production of IL-5 and IFN-γ, which are the indicator cytokines for Th1 and Th2 cells [36].

Lastly, we demonstrated that the mechanism of action of CaeA is quite distinct from immunosuppressive drug CsA. Since, CaeA does not follow the calcineurin pathway, therefore making it a unique and potent immunosuppressive molecule. Consequently, it has enough potential in future for treating autoimmune diseases, allergies and graft rejection.

## Supporting Information

Figure S1 CaeA production and its purification. (A) Micelle structure of *A. spitiensis* [200×]; (B) kinetics of the growth and production of CaeA by *A. spitiensis*; (C) TLC analysis of fractions obtained from High Performance Flash Chromatography (HPFC).
(TIF)

Figure S2 Structure elucidation of the isolated compound. ^13^C NMR spectrum of CaeA in DMSO, (75 MHz), full spectrum (Fig. 2.1A), expanded portion of spectrum A starting from 105 to 165 ppm (Fig. 2.1B); ^1^H NMR spectrum of CaeA in DMSO, (300 MHz) Full spectrum (Fig. 2.2A), expanded portion of spectrum A starting from 7.2 to 9.0 ppm (Fig. 2.2B); ^1^H NMR spectrum of CaeA in CDCl_3_, (300 MHz) (Fig. 2.3A), full spectrum, expanded portion of spectrum A starting from 7.2 to 8.8 ppm (Fig. 2.3B); ^1^H NMR spectrum of CaeA's methyl derivative in CDCl_3_ (300 MHz) (Fig. 2.4A), full spectrum, expanded portion of spectrum A starting from 7.1 to 8.9 ppm (Fig. 2.4B); Distortionless Enhancement by Polarization Transfer (DEPT), DEPT-90 (Fig. 2.5A), DEPT-135 experiments by using Bruker (300 MHz) (Fig. 2.5B); Mass spectrum of CaeA (Fig. 2.6), the molecular weight of CaeA is 229.09 by using Double focusing Mass spectrophotometer (VG,70S, 250). The structure of CaeA (Fig. 2 C).
(TIF)

Figure S3 CaeA does not affect the macrophage function. Peritoneal macrophages were stimulated with LPS and incubated for 48 h. (A) Flowcytometric histograms represent CD86 expression. ELISA data shows release of (B) TNF-α; (C) IL-6; (D) NO production by Griess method. Results are denoted as percentage (A); mean±SD (B–D). ‘ns’ stands for non-significant. Data are representative of 2–3 independent experiments.
(TIF)

Figure S4 CaeA administration in mice does not induce toxicity. Acute toxicity test was performed by feeding animals with indicated concentrations of CaeA. (A) Bar diagram represents the weight of animals at different time intervals. Dot plots depict the weight of (B) liver, (C) heart, (D) kidney, (E) spleen. Bar diagrams signify intake of (F) food; (G) water at different time intervals. Results are represented as mean±SD with 4–6 mice per group. ‘ns’ stands for non-significant.
(TIF)

Table S1 ^1^H and ^13^C NMR spectral data of Caerulomycin A (CaeA). ^1^H and ^13^C NMR were recorded on BruckerAvance300 (300 MHz for ^1^H; 75 MHz for ^13^C) spectrometer using CDCl_3_ or DMSO as solvent. Tetramethylsilane (TMS)/residual CHCl_3_ were used as internal standard. Chemical shifts δ are reported as downfield from TMS. Values of coupling constant *J* are reported in Hz.
(DOCX)
