# Supplementary figures and images for: Caerulomycin A Suppresses Immunity by Inhibiting T Cell Activity
Source: PLoS One. 2014 Oct 6;9(10):e107051. doi: 10.1371/journal.pone.0107051 (PMC4186789; doi:10.1371/journal.pone.0107051)

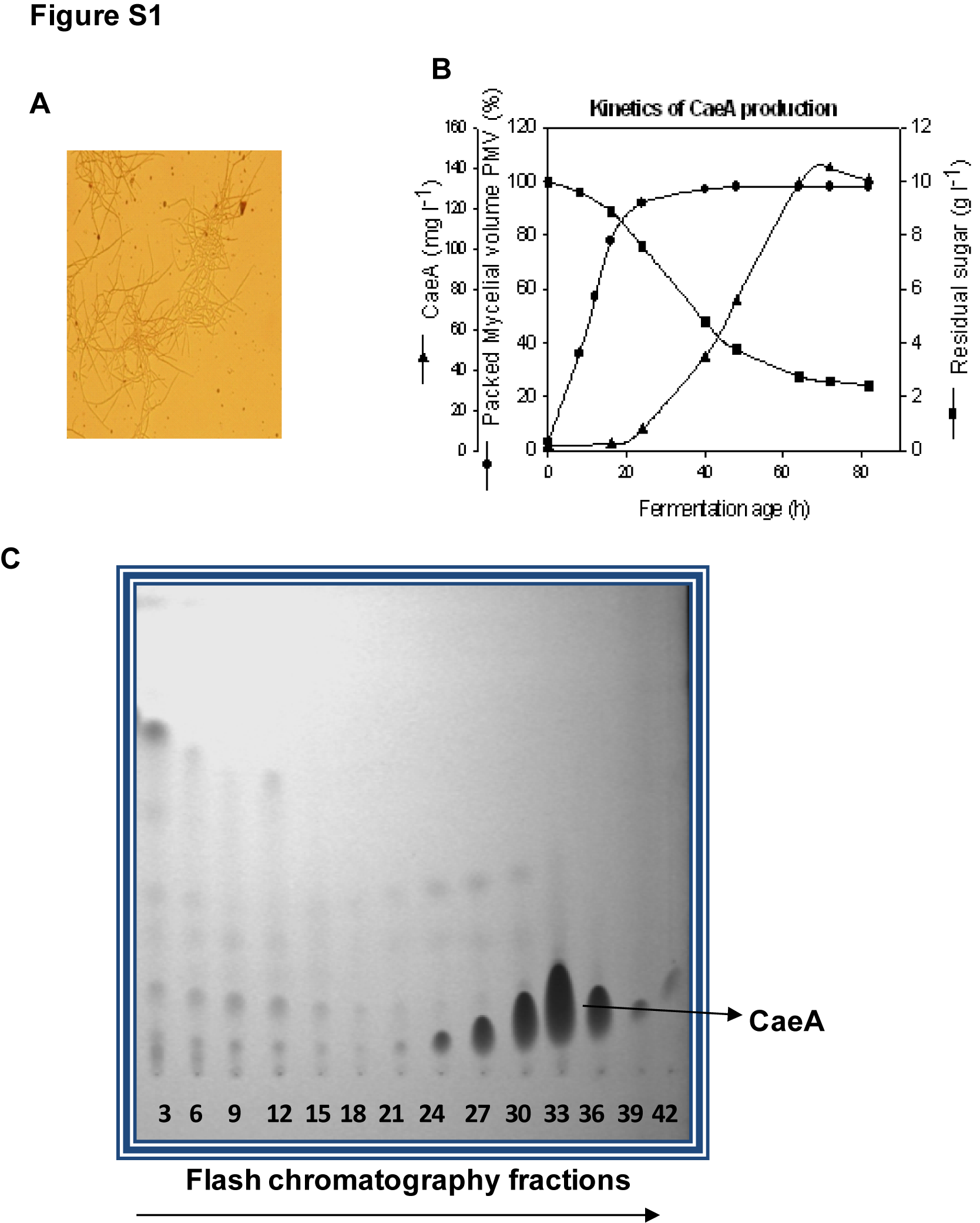

Supplement: Figure S1 — CaeA production and its purification. (A) Micelle structure of A. spitiensis [200×]; (B) kinetics of the growth and production of CaeA by A. spitiensis; (C) TLC analysis of fractions obtained from High Performance Flash Chromatography (HPFC). (TIF) [file pone.0107051.s001.tif]

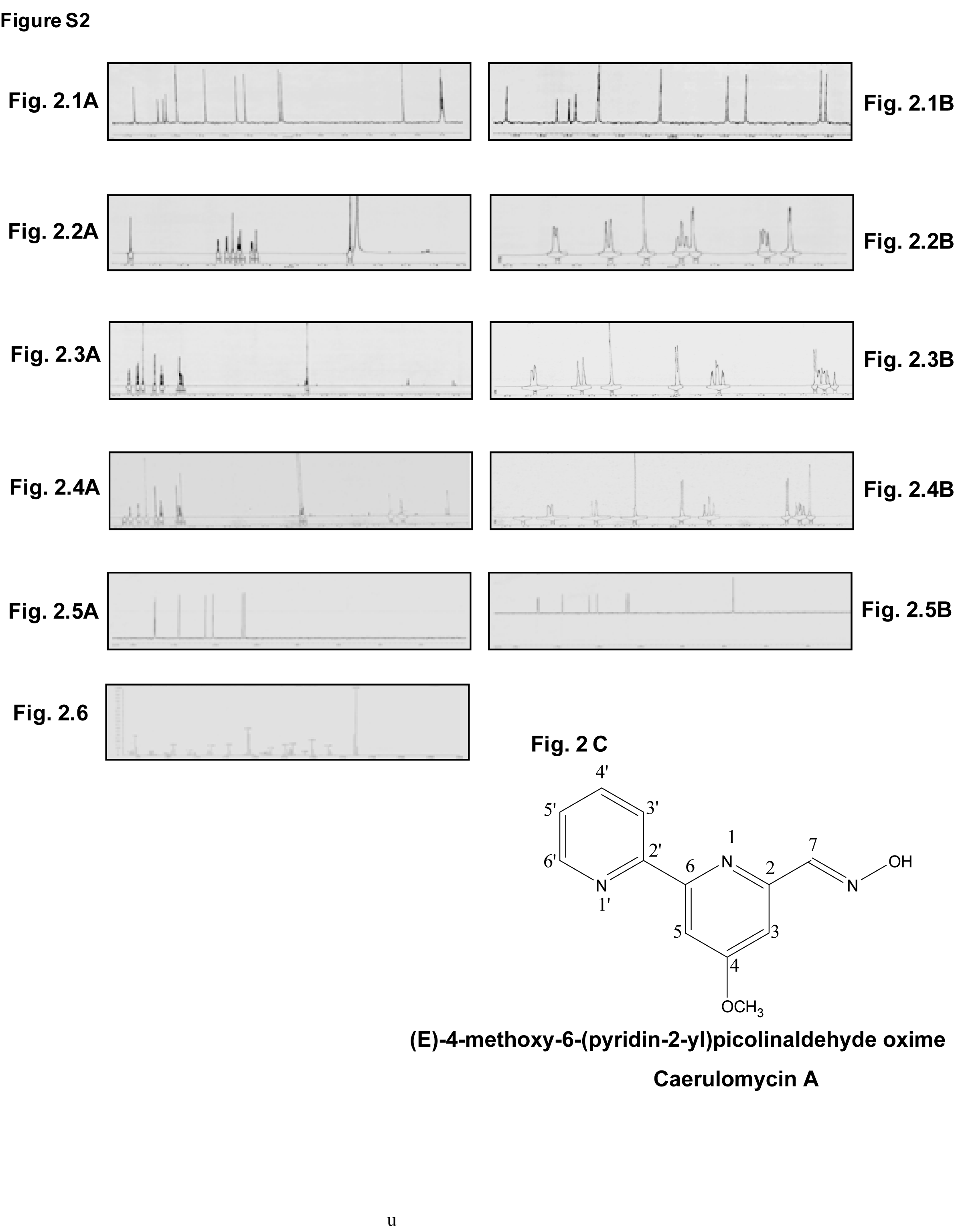

Supplement: Figure S2 — Structure elucidation of the isolated compound. 13C NMR spectrum of CaeA in DMSO, (75 MHz), full spectrum (Fig. 2.1A), expanded portion of spectrum A starting from 105 to 165 ppm (Fig. 2.1B); 1H NMR spectrum of CaeA in DMSO, (300 MHz) Full spectrum (Fig. 2.2A), expanded portion of spectrum A starting from 7.2 to 9.0 ppm (Fig. 2.2B); 1H NMR spectrum of CaeA in CDCl3, (300 MHz) (Fig. 2.3A), full spectrum, expanded portion of spectrum A starting from 7.2 to 8.8 ppm (Fig. 2.3B); 1H NMR spectrum of CaeA's methyl derivative in CDCl3 (300 MHz) (Fig. 2.4A), full spectrum, expanded portion of spectrum A starting from 7.1 to 8.9 ppm (Fig. 2.4B); Distortionless Enhancement by Polarization Transfer (DEPT), DEPT-90 (Fig. 2.5A), DEPT-135 experiments by using Bruker (300 MHz) (Fig. 2.5B); Mass spectrum of CaeA (Fig. 2.6), the molecular weight of CaeA is 229.09 by using Double focusing Mass spectrophotometer (VG,70S, 250). The structure of CaeA (Fig. 2 C). (TIF) [file pone.0107051.s002.tif]

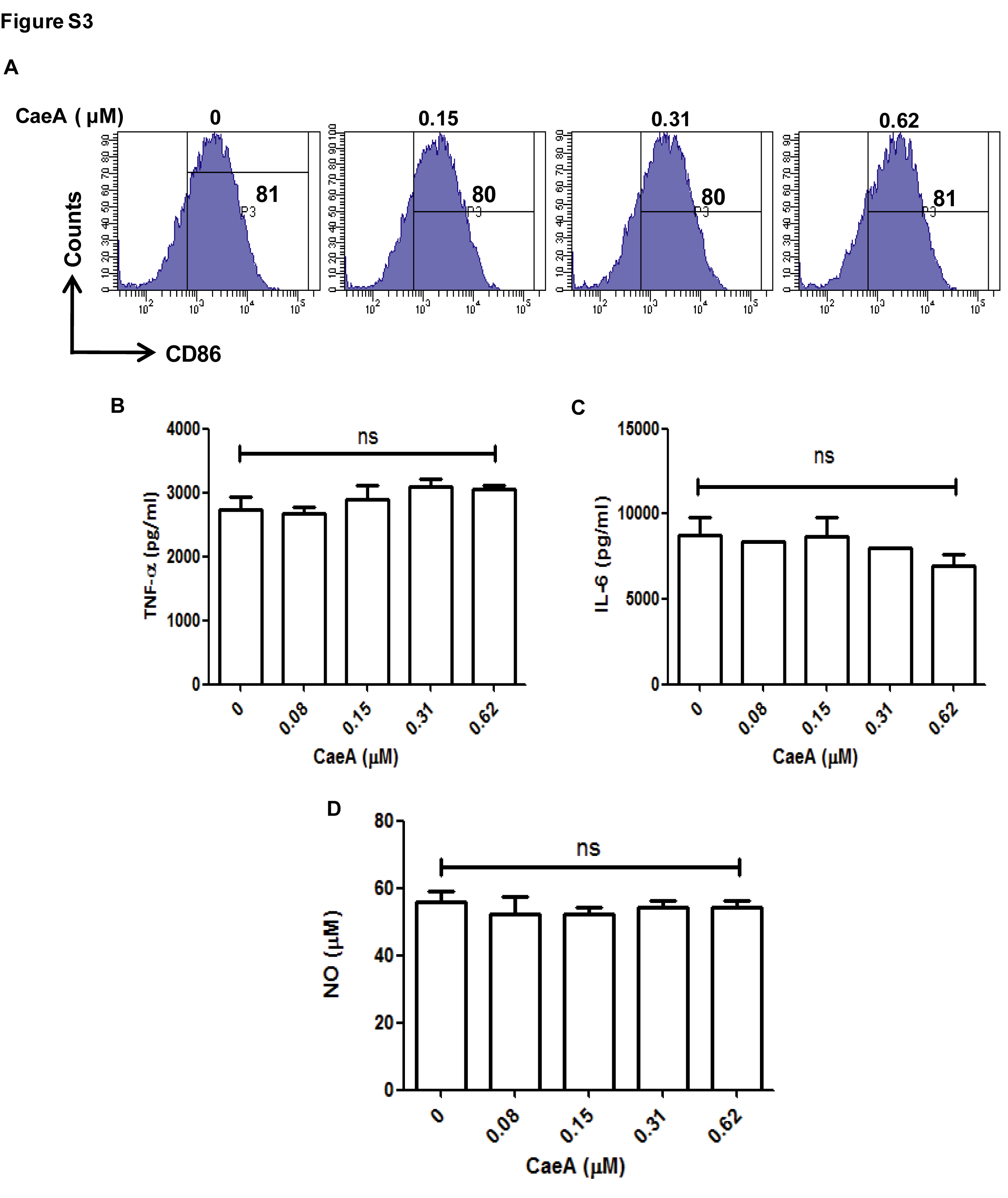

Supplement: Figure S3 — CaeA does not affect the macrophage function. Peritoneal macrophages were stimulated with LPS and incubated for 48 h. (A) Flowcytometric histograms represent CD86 expression. ELISA data shows release of (B) TNF-α; (C) IL-6; (D) NO production by Griess method. Results are denoted as percentage (A); mean±SD (B–D). ‘ns’ stands for non-significant. Data are representative of 2–3 independent experiments. (TIF) [file pone.0107051.s003.tif]

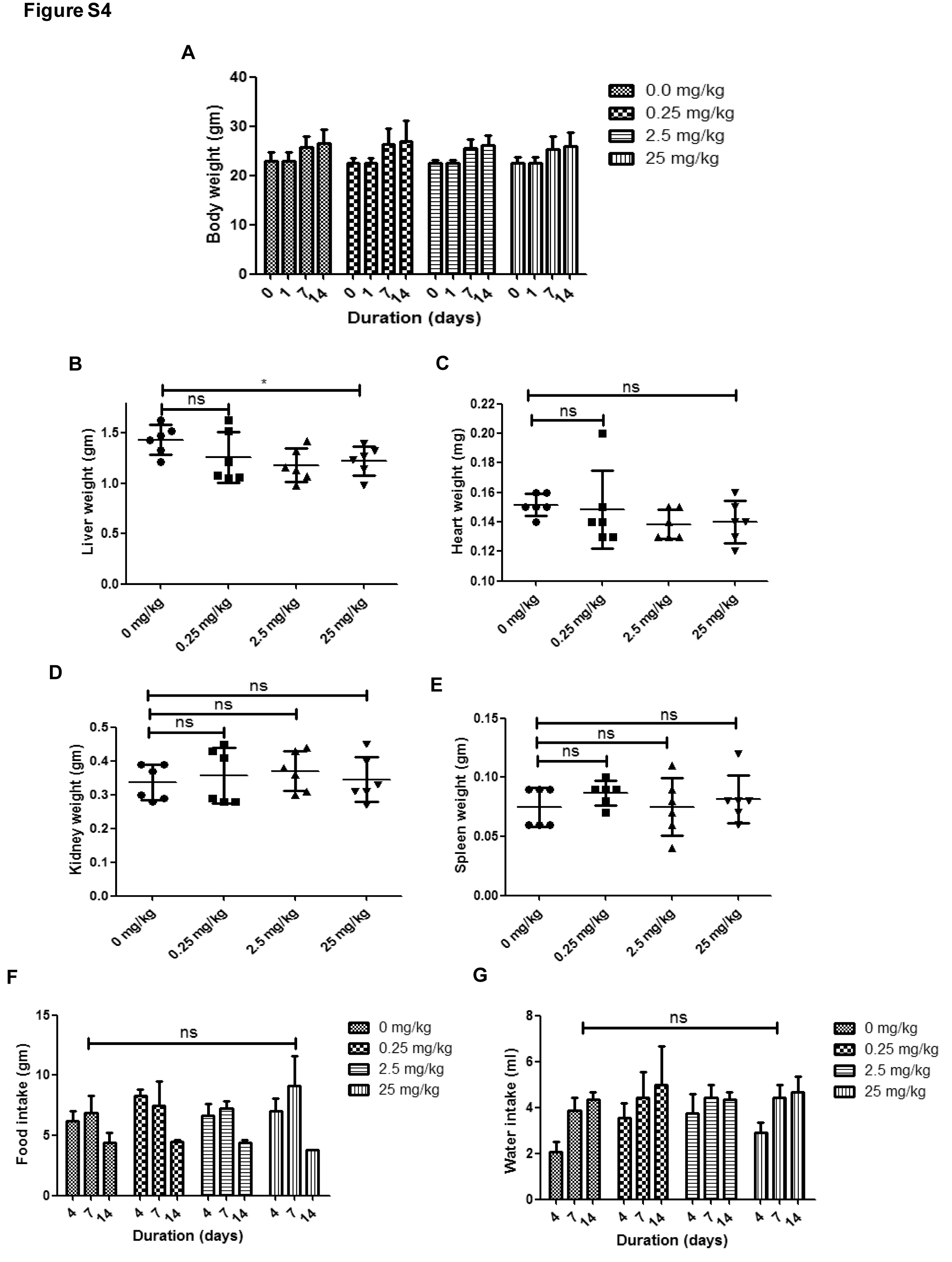

Supplement: Figure S4 — CaeA administration in mice does not induce toxicity. Acute toxicity test was performed by feeding animals with indicated concentrations of CaeA. (A) Bar diagram represents the weight of animals at different time intervals. Dot plots depict the weight of (B) liver, (C) heart, (D) kidney, (E) spleen. Bar diagrams signify intake of (F) food; (G) water at different time intervals. Results are represented as mean±SD with 4–6 mice per group. ‘ns’ stands for non-significant. (TIF) [file pone.0107051.s004.tif]
