# Supplementary material for: Caerulomycin A Suppresses Immunity by Inhibiting T Cell Activity
Source: PLoS One. 2014 Oct 6;9(10):e107051. doi: 10.1371/journal.pone.0107051 (PMC4186789; doi:10.1371/journal.pone.0107051)
Supplement: Table S1 — 1H and 13C NMR spectral data of Caerulomycin A (CaeA). 1H and 13C NMR were recorded on BruckerAvance300 (300 MHz for 1H; 75 MHz for 13C) spectrometer using CDCl3 or DMSO as solvent. Tetramethylsilane (TMS)/residual CHCl3 were used as internal standard. Chemical shifts δ are reported as downfield from TMS. Values of coupling constant J are reported in Hz. (DOCX) [file pone.0107051.s005.docx]

**Table S1. ^13^C NMR and ^1^H NMR spectral data of Caerulomycin A**

| Assignment* | CaeA (DMSO) | | CaeA (CDCI_3_) | CaeA O-Methyl  derivative (CDCI_3_) |
| --- | --- | --- | --- | --- |
|  | δC | δH | δH | δH |
| 2 | 156.8 |  |  |  |
| 3 | 106.4 | 7.92 (d, *J* = 2.16Hz) | 7.98 (d, *J* = 2.69 Hz) | 7.95 (d, *J* = 2.3 Hz) |
| 4 | 166.5 | - | - | - |
| 5 | 105.5 | 7.36 (d, *J* = 2.16 Hz) | 7.33 (d, *J* = 2.69 Hz) | 7.34 (d, *J* = 2.3 Hz) |
| 6 | 153.4 | - | - | - |
| 7 | 148.8 | 8.20 (s) | 8.30 (s) | 8.20 (s) |
| OCH_3_ | 55.5 | 3.97 (s) | 3.95 (s) | 3.96 (s) |
| 2’ | 154.5 | - | - | - |
| 3’ | 120.7 | 8.41 (d, *J* = 8.0 Hz) | 8.45 (d, *J* = 8.1 Hz) | 8.43 (d, *J* = 7.5 Hz) |
| 4’ | 137.2 | 7.96 (dd, *J* = 8.0 Hz, 7.2 Hz) | 7.80 (dd, *J* = 8.1 Hz, 6.8 Hz) | 7.81 (dd, *J* = 7.5 Hz, 6.0 Hz) |
| 5’ | 124.4 | 7.51 (dd, *J* = 7.2 Hz, 5.0 Hz) | 7.28 (dd, *J* = 6.8 Hz, 5.6 Hz) | 7.31 (dd, *J* = 6.0 Hz, 5.8) |
| 6’ | 149.2 | 8.72 (d, *J* = 5.0 Hz) | 8.67 (d, *J* = 5.6 Hz) | 8.67 (d, *J* = 5.8 Hz) |
| NOH/  NOCH_3_ |  | 11.8 (s) | - | 4.03 (s) |

*see Fig 2C for numbering. s, singlet; d, doublet

**IR spectral data in KBr**

2847, 2360, 1589, 1560, 1430, 1360, 1168, 1054, 981, 789, 741

**Mass spectra (Figure S2.6)**

M^+^ = 229.09

**Microanalysis Found** C=63.11, N=18.03, H=4.80. Calculated for C_12_H_11_N_3_O_2_ C=62.87, N=18.33, H=4.84
